# Immune checkpoint inhibitor-induced myocarditis: a comprehensive review with clinical case insights

**DOI:** 10.47487/apcyccv.v6i3.502

**Published:** 2025-09-24

**Authors:** María José Santa-Ana-Bayona, Camila Ponce-Acosta, Edgar Quispe-Silvestre, Gilberto H. Acosta-Gutiérrez, Alfonso González-Trejo, Hugo A. Valencia-Hernández, Enrique C. Guerra, Pablo O. Alarcón-Toxqui, Karina Martínez-Bañagas, Nilda Espinola-Zavaleta

**Affiliations:** 1 Facultad Mexicana de Medicina, Universidad La Salle, Ciudad de México, México. Universidad La Salle Facultad Mexicana de Medicina Universidad La Salle Ciudad de México Peru; 2 Departamento de Cardiología, Clínica Ricardo Palma, Lima, Perú. Universidad Ricardo Palma Departamento de Cardiología Clínica Ricardo Palma Lima Peru; 3 Instituto de Ciencias Biomédicas, Universidad Autónoma de Ciudad Juárez, Juárez, México Universidad Autónoma de Ciudad Juárez Instituto de Ciencias Biomédicas Universidad Autónoma de Ciudad Juárez Juárez Mexico; 4 Facultad de Medicina, Universidad Nacional Autónoma de México, Ciudad de México, México. Universidad Nacional Autónoma de México Facultad de Medicina Universidad Nacional Autónoma de México Ciudad de México Mexico; 5 Facultad de Medicina, Universidad Villa Rica, Veracruz, México. Universidad Villa Rica Facultad de Medicina Universidad Villa Rica Veracruz Mexico; 6 Programa (PECEM), Facultad de Medicina, Universidad Nacional Autónoma de México, Ciudad de México, México. Universidad Nacional Autónoma de México Programa (PECEM) Facultad de Medicina Universidad Nacional Autónoma de México Ciudad de México Mexico; 7 Facultad de Medicina, Benemérita Universidad Autónoma de Puebla, Puebla, México. Benemérita Universidad Autónoma de Puebla Facultad de Medicina Benemérita Universidad Autónoma de Puebla Puebla Mexico; 8 Facultad de Ciencias de la Salud Valle de las Palmas, Universidad Autónoma de Baja California, Tijuana Baja California, México. Universidad Autónoma de Baja California Facultad de Ciencias de la Salud Valle de las Palmas Universidad Autónoma de Baja California Tijuana Baja California Mexico; 9 Departamento de Cardiología Nuclear, Instituto Nacional de Cardiología Ignacio Chávez, Ciudad de México, México. Departamento de Cardiología Nuclear Instituto Nacional de Cardiología Ignacio Chávez Ciudad de México México; 10 Departamento de Ecocardiografía, Centro Médico ABC, Ciudad de México, México. Departamento de Ecocardiografía Centro Médico ABC Ciudad de México México

**Keywords:** Immune Checkpoint Inhibitors, Myocarditis, Cardiotoxicity, Cardiac Imaging, Inhibidores de Punto de Control Inmunológico, Miocarditis, Cardiotoxicidad, Técnicas de Imagen Cardiaca

## Abstract

Immune checkpoint inhibitors (ICIs) have revolutionized cancer therapy by significantly improving long-term outcomes across multiple malignancies. Despite their benefits, ICIs can lead to immune-related adverse events, including rare but severe cardiovascular toxicity such as myocarditis, which can be life-threatening. This comprehensive review aims to explore and discuss the mechanisms, clinical presentation, diagnostic challenges, and management strategies of ICI-induced myocarditis, emphasizing the need for early detection and timely intervention. As the use of ICIs continues to expand, further research is essential to fully elucidate the underlying mechanisms and optimize therapeutic strategies to mitigate this potentially fatal complication while maintaining the efficacy of cancer therapy.

## RESUMEN

Los inhibidores del punto de control inmunológico (IPCI) han revolucionado la terapia contra el cáncer al mejorar significativamente los resultados a largo plazo en múltiples tipos de neoplasias. A pesar de sus beneficios, pueden ocasionar eventos adversos relacionados con el sistema inmunitario, entre ellos la toxicidad cardiovascular, poco frecuente pero grave, como la miocarditis, una enfermedad potencialmente mortal. Esta revisión integral tiene como objetivo explorar y discutir los mecanismos, la presentación clínica, los retos diagnósticos y las estrategias de manejo de la miocarditis inducida por los inhibidores del punto de control inmunológico, enfatizando la importancia de la detección temprana e intervención oportuna. A medida que se expande el uso de los IPCI, es esencial continuar con la investigación para elucidar completamente los mecanismos subyacentes y optimizar las estrategias terapéuticas, con el fin de mitigar esta complicación potencialmente fatal sin comprometer la eficacia del tratamiento oncológico.

## Introduction

**Figure 1:**
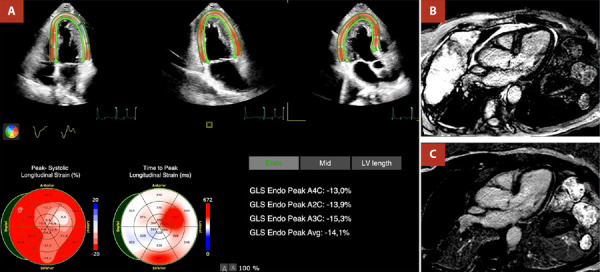
Transthoracic echocardiogram and cardiac magnetic resonance in a patient with ICI cardiotoxicity. **A)** Transthoracic echocardiogram revealing decreased left ventricular global longitudinal strain (-14.1%). **B)** and **C)** Cardiac magnetic resonance in T1-weighted inversion-recovery sequence demonstrating mid anteroseptal late gadolinium enhancement, consistent with myocarditis.

An 80-year-old man with newly diagnosed programmed death ligand 1 (PD-L1) - positive (40%) non-small cell lung cancer received guideline‐directed sequential therapy comprising platinum‐based chemotherapy followed by pembrolizumab. Baseline echocardiography demonstrated preserved left ventricular ejection fraction (LVEF) and normal cardiac biomarkers. Prior to the fourth pembrolizumab infusion, he developed dysarthria and progressive bilateral lower‐limb weakness. Laboratory evaluation revealed elevated NT-proBNP and troponin, and echocardiography showed a decline in LVEF (48%) with impaired global longitudinal strain (-14%). Cardiac magnetic resonance imaging (MRI) confirmed non-ischemic late gadolinium enhancement consistent with myocarditis (Figure 1). High‐dose methylprednisolone was initiated, resulting in marked recovery of neuromuscular strength and cardiac function. 

Cancer remains a leading global health challenge. Despite the complexity of cancer, advancements in therapeutic strategies such as chemotherapy, hormonal treatments, and gene-targeted therapies have significantly improved survival rates. Among these, a major breakthrough in the past decade has been the introduction of immune checkpoint inhibitors (ICIs), which have revolutionized cancer treatment. Dr. James P. Allison’s pioneering work laid the foundation for these therapies, fundamentally changing the approach to cancer care. ^(^^1^

ICIs are monoclonal antibodies that target specific proteins, such as cytotoxic T-lymphocyte-associated protein 4 (CTLA-4), programmed death-1 (PD-1), and its ligand PD-L1.^2^ These proteins play a crucial role in helping cancer cells evade the immune system. Inhibiting these checkpoints, ICIs enhance the body’s ability to mount a strong anti-tumor immune response. However, this heightened immune activity can also trigger adverse reactions known as immune-related adverse events (irAEs), affecting various organs and systems. While most irAEs involve the skin, gastrointestinal tract, liver, pulmonary, and endocrine systems, cardiovascular toxicity, including myocarditis, remains rare but potentially fatal. ^3^^,^^4^

Cardiovascular irAEs, although rare (<1% of patients), are particularly worrisome due to their potentially severe and life-threatening nature. ^5^ These events encompass a wide range of conditions, including myocarditis, acute coronary syndromes, congestive heart failure, non-malignant pericardial disorders, dysrhythmias, and cardiac arrest. ^6^^,^^7^^)^ Among these, myocarditis stands out as a critical and often underrecognized complication, necessitating heightened awareness and prompt intervention. In addition, given its diverse presentation and the overlap with other cardiovascular conditions, early recognition is crucial, as delayed diagnosis can result in fatal outcomes, including fulminant heart failure.

## Mechanism of checkpoint inhibitors

Evasion of the immune system is a hallmark of cancer, allowing tumor cells to proliferate and metastasize unchecked. ICIs offer a therapeutic strategy by targeting pathways that tumors exploit to escape immune detection. These drugs work by inhibiting specific immune checkpoints, which typically function as “brakes” of the immune system to prevent overactivation. By releasing these inhibitory pathways, ICIs reinvigorate T-cell function, enhancing their capacity to effectively target tumor cells. ^8^ The primary targets of ICIs are the CTLA-4 and the PD-1 pathways, both of which play distinct but complementary roles in regulating immune responses. ^8^

*CTLA-4*

**Figure 2:**
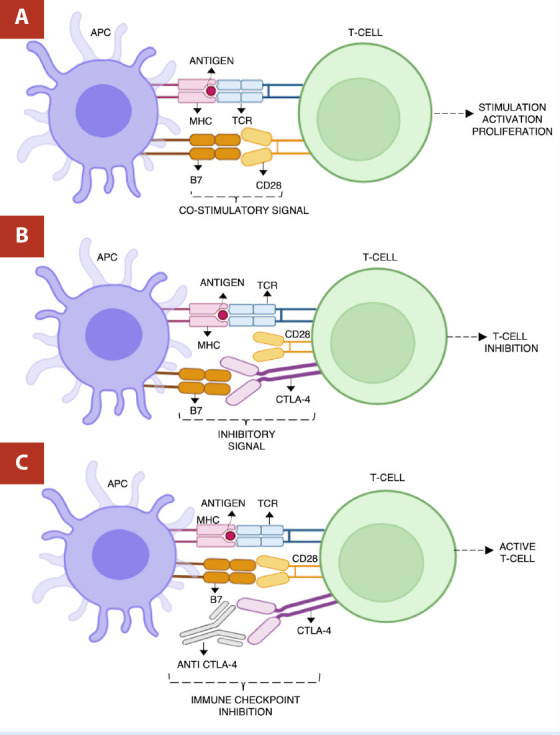
Anti CTLA-4 mechanism of action. A) CD28 on T-cell binding to B7 on antigen-presenting cell produces stimulation, activation, and proliferation of T-cell response. B) CTLA-4 binding to B7 and blocking T-cell co-stimulation mediated by CD28, resulting in inhibition and suppression of T-cells. C) Anti CTLA-4 binding CTLA-4, allowing CD28-mediated signaling to stimulate T-cells.

CTLA-4 is a receptor expressed on T-cells that binds with B7 molecules on antigen-presenting cells (APCs). It competes with the co-stimulatory receptor CD28; however, it has a much higher affinity than the latter. ^9^ By binding to B7, CTLA-4 blocks the co-stimulation of T cells mediated by CD28, transmitting an inhibitory signal that suppresses T-cell activation and limits the immune response. Inhibitors like ipilimumab block CTLA-4, preventing its binding to B7 and enhancing T-cell activation and proliferation by allowing CD28-mediated signaling to proceed unimpeded. ^9^^)^ (Figure 2)

*Created with Biorender*

*PD-1/PD-L1*

**Figure 3:**
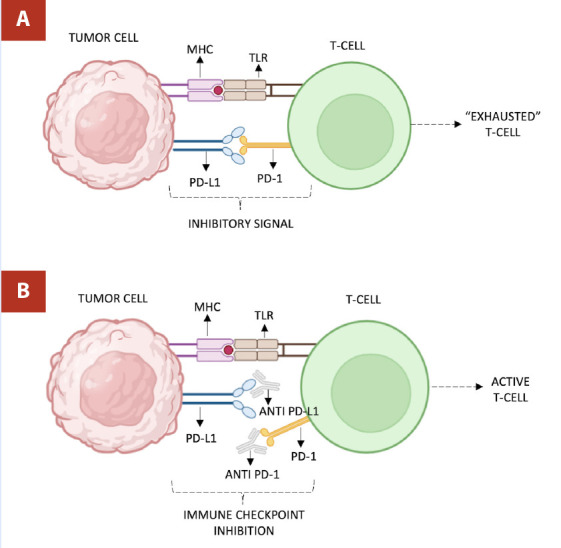
Anti PD-1/PD-L1 mechanism of action. **A)** PD-1 on T-cell binding to its ligand PD-L1 on tumor, transmitting an inhibitory signal resulting in “exhausted T-cell” phenotype. **B)** Inhibitors targeting PD-1 and PD-L1 block interaction, restoring T-cell activation.

PD-1 is an inhibitory receptor expressed on T-cells. When PD-1 binds to its ligands PD-L1 or PD-L2, which can be expressed on tumor cells and APCs, it transmits an inhibitory signal that dampens T-cell activity, helping tumors evade immune detection. Inhibitors such as pembrolizumab and nivolumab (targeting PD-1), and atezolizumab (targeting PD-L1) block these interactions, restoring T-cell activity and enabling a more robust response against the tumor. ^10^^)^ (Figure 3)

*Created with Biorender*

*Combination therapy*

Complementary roles of CTLA-4 and PD-1/PD-L1 inhibitors have been proposed under the rationale that blocking both pathways may yield synergistic effects by enhancing T-cell activation at multiple stages of the immune response. CTLA-4 blockade facilitates initial T-cell activation, while PD-1/PD-L1 inhibition prevents T-cell exhaustion within the tumor microenvironment. ^11^^)^ As a result, dual blockade can generate a more potent anti-tumor response than either agent alone. However, this approach carries a notable risk of severe immune-related toxicities, as enhancing immune activation on multiple fronts increases the likelihood of off-target autoimmune effects. In addition, combination therapy has been associated with higher rates of treatment discontinuation due to adverse events, necessitating a careful risk-benefit analysis for each patient. ^12^

## Epidemiology

*Demographic Factors*

ICI-induced myocarditis exhibits certain demographic trends, with a notable male predominance. A recent study by Qin *et al*.^13^^)^ reported that 77.4% of patients were male, with a mean age of 65 years. Similarly, another study indicated a mean age of 62 years. ^14^^)^ Notably, myocarditis has been observed to occur with higher frequency in certain types of cancer such as melanoma, lung cancer, renal cell carcinoma, or other types of rare malignancies such as thymic epithelial tumors. ^6^

*Incidence and Mortality*

Incidence ranges from 0.06 to 1.14% among patients receiving ICI therapy, with reported mortality rates varying between 24% to 50%. ^15^^,^^16^^)^ A meta-analysis conducted from a pharmacovigilance database highlighted that among irAEs, myocarditis had the highest fatality rate with 52 out of 131 reported cases (39.7%) resulting in death, underscoring the critical need for early recognition and treatment. ^4^

*Monotherapy vs combination therapy*

The risk of developing ICI-induced myocarditis appears to be higher in patients receiving combination therapy compared to monotherapy. For instance, Johnson et al. reported an incidence of 0.27% with single-agent ICIs, while Naqash *et al*. found a slightly lower incidence of 0.15% with monotherapy. ^7^^,^^17^ In contrast, the risk is increased with combination regimens: Mahmood et al. observed an incidence of 1.14%, and Naqash et al. reported an incidence of 0.36% in patients undergoing combination therapy. ^7^^,^^14^ Another research suggests that the use of combined immunotherapy does not markedly raise the incidence of severe cardiac complications; nonetheless, the available evidence is limited and lacks sufficient robustness to support definitive conclusions. ^18^

*Time of onset*

Myocarditis typically manifests early in the course of treatment. A multicenter study found that the median time to onset of myocarditis from first ICI was 34 days after the initial ICI dose, with 81% of cases presenting within three months of starting therapy. These findings are consistent with another study that reported a median onset time of 6.3 weeks. ^13^^,^^14^

## Pathophysiology of ICI-induced myocarditis

ICI-induced myocarditis is thought to result primarily from autoimmune mechanisms, although the exact pathophysiology remains incompletely defined. One central hypothesis suggests that non-specific T-cell activation promotes CD8+ T-cell infiltration into myocardial tissue, potentially through cross-reactivity with shared tumor and cardiac antigens, recognition of homologous muscle antigens, or T-cell receptors targeting unrelated antigens that induce myocardial inflammation.^12^^,^^17^^,^^19^^)^ Additionally, CD8+ T cells may directly target α-myosin, leading to myocardial injury. Macrophages also appear to play a significant role, as T-cell-derived IFN-γ promotes the expansion of pro-inflammatory macrophage populations expressing chemokines such as CCR2, CXCL9, and CXCL10. Furthermore, chemokine and cytokine signaling pathways contribute to disease progression, with receptors like CCR5 and CXCR3 facilitating immune cell recruitment and amplifying inflammation via T-cell-macrophage interactions. ^20^

## Clinical presentation

IrAE may involve nearly all organ systems, among the most frequent are colitis (more prevalent with CTLA4-targeted agents), thyroiditis, hypophysitis, primary adrenal insufficiency, insulin deficient diabetes mellitus, immune-mediated pneumonitis or sarcoid-like reactions (more frequent with inhibitors of the PD1 or PD-L1 pathway), inflammatory arthritis, vasculitis, autoimmune hemolytic anemia, immune thrombocytopenia, and neutropenia, myocarditis, pericarditis, or conduction abnormalities, acute interstitial nephritis, myositis, myasthenia gravis, Guillain-Barré-like syndromes and autoimmune encephalitis, with potential overlap syndromes. Although many of these toxicities reflect a robust immune activation, their unpredictable onset, variable severity, and frequently nonspecific clinical presentation pose significant diagnostic and therapeutic challenges. ^21^

Cardiovascular IrAE should be suspected in the presence of any new cardiovascular symptom or sign, electrocardiographic changes, or cardiac biomarker elevation. Patients may present with a variable range of symptoms from mild fatigue and dyspnea to severe fulminant cases that can present with cardiogenic shock, complete atrioventricular block, ventricular arrhythmias, acute heart failure, and cardiac arrest. ^22^

A classification system has been proposed by the American Society of Clinical Oncology (ASCO) clinical practice guidelines. **(**Table 1**)** Grade 1: asymptomatic patients with abnormal cardiac biomarkers or ECG abnormalities. Grade 2: abnormal cardiac biomarker testing, mild symptoms, or new ECG abnormalities (no conduction delay). Grade 3: abnormal cardiac biomarkers with moderate symptoms or new conduction delay, and grade 4: moderate-severe decompensation, life-threatening disease, and IV medication or intervention required. ^23^

**Table 1:** Immune checkpoint inhibitor-induced myocarditis severity scale. American Society of Clinical Oncology grading system for cardiovascular toxicities. ^5^

GRADE 1	GRADE2	GRADE 3	GRADE 4
Asymptomatic patients + Abnormal cardiac biomarkers and Normal ECG	Abnormal cardiac biomarkers + Mild symptoms or new ECG abnormalities (no conduction delay)	Moderate symtoms + Abnormal cardiac biomarkers or new conduction delay	Life-threatening disease, moderate-severe decompensation + Requirement of IV medication or intervention
HOLD ICI			
* Reassess troponin 6 hours later		* Discontinue ICI	* Discontinue ICI
* May consider rechallenge		* High-dose corticosteroids (Early initiation)	* High-dose corticosteroids (Early initiation)
		* Patients without immediate response: consider cardiac transplant rejection doses of corticosteroids (methylprednisolone 1 g / day) + addition of mycophenolate, infliximab or antithymocyte globulin	* Patients without immediate response: consider cardiac transplant rejection doses of corticosteroids (methylprednisolone 1 g / day) + addition of mycophenolate, infliximab or antithymocyte globulin
		* Life-threatening cases: consider abatacept or alemtuzumab (as additional immunosuppression)	* Life-threatening cases: consider abatacept or alemtuzumab (as additional immunosuppression)

Abbreviations: ECG: electrocardiogram; ICi: Immune Checkpoint Inhibitor; IV: intravenous

Noteworthy, symptoms of non-cardiovascular irAE may be prominent like those of myositis or myasthenia gravis. Studies have shown that myocarditis and myositis frequently co-occur, and patients with fatal outcomes had concurrent or preceding myositis, suggesting a high-risk population where myositis might indicate a poor prognosis. ^4^^,^^7^ Clinically, myocarditis may manifest with nonspecific symptoms such as chest discomfort, exertional dyspnea, arrhythmias, or may remain asymptomatic and be detected only through elevated cardiac biomarkers. In contrast, myositis commonly presents with proximal muscle weakness, myalgia, and markedly elevated creatine kinase levels. Notably, several patients developed overlap syndromes that included features of both myocarditis and myositis, occasionally accompanied by myasthenia gravis. These overlap presentations were associated with higher clinical severity and worse prognosis. ^24^

## Diagnosis

Currently, there are no standard tests or studies for the diagnosis of ICI-associated myocarditis. However, a combination of clinical evaluation, patient history, cardiac biomarkers, imaging studies, and sometimes endomyocardial biopsy is useful. Importantly, diagnostic testing should aim to rule out other more common causes of the clinical presentation and test abnormalities.

**Figure 4:**
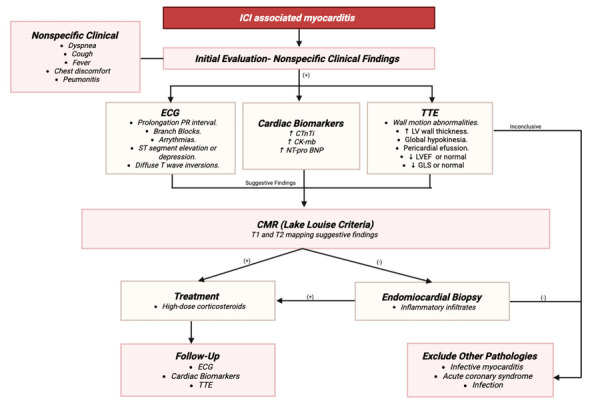
Diagnostic approach for suspected immune checkpoint inhibitor-associated myocarditis. Structured diagnostic algorithm for suspected myocarditis associated with immune checkpoint inhibitors. The approach integrates clinical evaluation, electrocardiography, cardiac biomarkers, and echocardiography as initial steps. Cardiac magnetic resonance imaging is recommended for further characterization, particularly using modified Lake Louise criteria. In cases of diagnostic uncertainty or hemodynamic compromise, endomyocardial biopsy may be warranted. The algorithm highlights a multimodal strategy to support early diagnosis and guide timely therapeutic intervention.

Work-up and evaluation should include electrocardiogram (ECG), troponin, creatine kinase (CK), BNP, and transthoracic echocardiogram. Additional testing guided by the cardiology team may include stress test, cardiac catheterization, and cardiac MRI.^5^^)^ Diagnosis relies heavily on clinical suspicion, biomarker elevation, and supportive imaging, although echocardiography and cardiac MRI may not always detect early changes. Awareness of these toxicities and early multidisciplinary intervention are essential to reduce associated morbidity and mortality. ^2^^)^ (Figure 4)

*Electrocardiogram (ECG)*

Approximately 90% of patients with ICI-induced myocarditis present with electrocardiographic abnormalities. ^25^ Common findings include prolongation of the PR interval, bundle branch blocks, and new-onset arrhythmias, including supraventricular and ventricular arrhythmias, and atrioventricular block. ST segment elevation or depression, and diffuse T wave inversions are also frequently observed. ^26^^)^ However, these electrocardiographic changes are non-specific, and other potential causes of these abnormalities should be thoroughly ruled out. Importantly, a normal ECG does not exclude the possibility of myocarditis, making clinical suspicion and further diagnostic evaluation essential.

*Cardiac Biomarkers*

Cardiac biomarkers play a pivotal role in both the diagnosis and prognostication of ICI-induced myocarditis. Serum troponin levels are the most widely used biomarkers that should raise the suspicion of myocarditis. Cardiac Troponin T (cTnT) appears to be more sensitive in the context of ICI-induced myocardial injury compared to troponin I (cTnI). A study conducted by Lehmann, et al. found that cTnT was elevated in 98% of patients, whereas cTnI was elevated in 88%. ^27^^)^ Troponin T is also often elevated in cases of concomitant myositis, accompanied by significant elevations of CK and its isoforms. ^28^ This makes cTnI potentially more specific for myocardial injury, as it is less frequently elevated in non-cardiac conditions.

According to the pharmacovigilance study conducted by Oliveria et al., elevated cardiac troponin levels were observed in over 90% of patients with myocarditis, while creatine kinase was significantly increased in nearly all individuals diagnosed with myositis. These findings highlight the central role of cardiac and muscle biomarkers in the early identification and diagnostic evaluation of these immune-mediated complications. ^2^^)^ Natriuretic peptides (B-type natriuretic peptide and N-terminal Pro-B-type natriuretic peptide [NT-proBNP]) are non-specific for myocarditis but can aid in the diagnosis of heart failure and are often elevated in the setting of ICI-related myocarditis. ^27^^,^^28^

Overall, both cTnT and cTnI are valuable biomarkers for diagnosing ICI-induced myocarditis. Additionally, obtaining CK and CK-MB can provide relevant clinical information, as elevations in these markers have been linked to the development and severity of myocarditis and may suggest the coexistence of ICI-induced myositis. ^28^^,^^29^

*Echocardiography*

Echocardiography represents the first line of imaging when myocarditis is suspected, given the non-invasive, non-ionizing, and bedside-available nature of the study. Suggestive findings include segmental wall motion abnormalities, increased LV wall thickness, global hypokinesia, and pericardial effusion. ^30^

Transthoracic echocardiography (TTE) may show a preserved ejection fraction, reduced ventricular function; findings such as regional wall motion abnormalities or diastolic dysfunction may also be present. However, in several cases, a normal LVEF does not rule out myocarditis despite ongoing myocardial injury, as cardiac function is normal in about 51% of patients and 38% of those who develop major adverse cardiac events (MACEs). ^24^^,^^25^

Global longitudinal strain (GLS) has emerged as a sensitive marker for myocardial dysfunction. Studies suggest that it can predict subclinical cardiac toxicity in patients with ICIs, and reductions in GLS can be detected even in the presence of a normal LVEF. ^30^^,^^31^ Moreover, a decrease in GLS has been associated with an increased risk of MACEs.^31^

*Cardiac Magnetic Resonance*

Cardiac magnetic resonance (CMR) may provide insight into myocardial inflammation and tissue characterization, edema, or fibrosis. Validated techniques to diagnose myocarditis with CMR include T2-weighted imaging, late gadolinium enhancement, extracellular volume fraction, T1-mapping and T2-mapping.^15^

Despite lacking specific findings on CMR for ICI-induced myocarditis, the Lake Louise Criteria for CMR diagnosis of myocarditis were recently updated to incorporate T1 and T2 mapping and are commonly used as they enhance the diagnostic accuracy for myocarditis in the context of ICI therapy. ^32^^)^ Although regarded as a key tool for evaluating myocardial inflammation, CMR has demonstrated variable sensitivity. While some patients exhibited late gadolinium enhancement and myocardial edema on T2-weighted sequences, a substantial proportion had nondiagnostic findings, suggesting a limitation of cardiac MRI in detecting immune-mediated myocarditis, particularly in early or mild presentations. ^24^

In the setting of clinically suspected acute myocarditis, CMR findings are consistent with myocarditis if both T1 and T2-based criteria are present. ^33^ Supportive criteria are also included and are not sufficient to support a diagnosis of myocarditis but are commonly identified in patients with myocarditis.

In the observational study conducted by Wang *et al*., CMR revealed considerable variability in diagnostic findings. Specifically, only 48% of patients exhibited late gadolinium enhancement, while merely 27% met the Lake Louise criteria for myocarditis. These results suggest that CMR may have limited sensitivity in the context of immune checkpoint inhibitor-associated myocarditis, emphasizing the necessity of integrating additional diagnostic modalities to ensure timely and accurate detection. ^4^

*Main CMR criteria:*

• T2-based imaging with one or more of the following signs of myocardial edema:

- ^o^Regional (an area of at least 10 contiguous pixels) high T2 signal intensity (SI).
- ^o^Global T2 SI ratio ≥2.0 in T2-weighted (T2W) CMR images.

*Regional or global increase of myocardial T2 relaxation time*

• T1-based imaging with either or both of the following findings of hyperemia or capillary leakage:

- ^o^Regional or global increase in native myocardial T1 relaxation time or extracellular volume (ECV).
- ^o^Areas with high SI in a nonischemic distribution pattern in LGE images (i.e., typically involving the subepicardium or midwall with infrequent involvement of subendocardium).

*Supportive criteria:*

• Pericardial effusion in cine CMR images.
• High SI of the pericardium in LGE, T1-mapping, or T2-mapping.
• T1 to T2 mapping systolic LV wall motion abnormality in cine CMR images.

*FDG-PET / CT*

In the context of ICI-induced myocarditis, the utility of fluorodeoxyglucose positron emission tomography combined with computed tomography (FDG-PET/CT) and coronary computed tomography angiography (CCTA) is considered limited and primarily reserved for selected clinical circumstances. These imaging modalities are not routinely incorporated into the standard diagnostic algorithm for this condition. However, they may hold diagnostic value when alternative etiologies are being considered.

Specifically, FDG-PET/CT can aid in detecting granulomatous or inflammatory myocardial involvement and may be useful when there is a need to differentiate between immune-mediated myocarditis and other forms of myocardial inflammation. On the other hand, CCTA serves as a non-invasive method for excluding obstructive coronary artery disease in patients presenting with chest pain or elevated cardiac biomarkers, particularly when ischemic heart disease remains a relevant differential diagnosis. Despite these potential applications, the routine use of either FDG-PET/CT or CCTA in the diagnosis of ICI-related myocarditis is not yet standard of care. This is primarily due to their limited specificity in identifying immune-mediated myocardial injury and the lack of validated imaging criteria for this clinical entity. Consequently, their implementation should be individualized, guided by the overall clinical context, and considered in cases where conventional imaging modalities such as echocardiography or cardiac MRI yield inconclusive findings or when alternative diagnoses must be systematically excluded. ^34^

*Role of Endomyocardial Biopsy*

Endomyocardial biopsy (EMB) remains the gold standard for diagnosing myocarditis, as it provides histopathological confirmation by identifying the presence of inflammatory infiltrates, including lymphocytes and macrophages. ^15^

EMB should be considered in cases of diagnostic uncertainty, in unstable patients, and in those who do not respond to initial therapy. However, if clinical suspicion of myocarditis is high, treatment should not be delayed. ^5^^)^ Its application is particularly valuable in complex clinical contexts requiring histopathological confirmation. To enhance diagnostic accuracy and reduce the risk of sampling error, it is recommended that the procedure be conducted at specialized centers with appropriate expertise, ensuring that multiple tissue samples are collected during the intervention. ^34^

A definition of ICI-induced myocarditis has been recently proposed by the International Cardio-Oncology Society, which considers either histopathological or clinical diagnosis, based on major and minor criteria. ^35^^)^ (Table 2)

**Table 2:** Immune checkpoint inhibitor-induced myocarditis definition by the International Cardio-Oncology Society. ^35^

ICI-Induced Myocarditis (International Cardio-Oncology Society Definition)	
Diagnosis: Histopathological or clinical diagnosis	
Histopathological diagnosis: Multifocal inflammatory cell infiltrates with overt cardiomyocyte loss by light microscopy	
Clinical diagnosis: cTn elevation (new or significant change from baseline) with 1 major criterion or 2 minor criteria, after exclusion of acute coronary syndrome and acute infectious myocarditis based on clinical suspicion		
Major criteria: CMR diagnostic for acute myocarditis (modified Lake Louise criteria)	Minor criteria: Clinical syndrome* * Ventricular arrhythmia (including cardiac arrest) and/or new conduction system disease* Decline in LV systolic function, with or without regional wall motion abnormalities in a non-Takotsubo pattern* Other immune-related adverse events, particularly myositis, myopathy, myasthenia gravis* Suggestive CMR

*Any one of the following: chest pain, palpitations, syncope, shortness of breath, orthopnea, diplopia, ptosis, fatigue, myalgias, lower-extremity oedema, light-headedness, dizziness, muscle weakness, cardiogenic shock

Abbreviations: cTn Cardiac troponin; CMR: cardiac magnetic resonance; LV: left ventricle

## Management

Management of ICI-induced myocarditis involves prompt cessation of the ICI and initiation of immunosuppressive therapy to mitigate and prevent further immune-mediated damage. High-dose corticosteroids (1-2 mg/kg/day of prednisone or equivalent) are the mainstay of treatment, followed by a 4-6 week gradual taper upon symptom and biomarker improvement. ^9^^,^^23^^)^ For patients who do not immediately respond to corticosteroids, escalation to cardiac transplant rejection doses of steroids (methylprednisolone 1 g every day) is recommended, along with the addition of other immunosuppressive agents such as mycophenolate mofetil, infliximab, or abatacept. Unfortunately, approximately 50% of patients may not respond to corticosteroid therapy and will need a second-line treatment strategy. ^36^

In the research conducted by Wang et al., the authors emphasize that high-dose systemic corticosteroids constitute the primary therapeutic approach for ICI-associated myocarditis and myositis. The early administration of intravenous methylprednisolone, typically followed by a gradual tapering regimen of prednisone, was frequently associated with favorable clinical responses. In cases characterized by fulminant progression or inadequate response to corticosteroids alone, escalation to additional immunosuppressive therapies is also considered a therapeutic approach. These included intravenous immunoglobulin, mycophenolate mofetil, and agents targeting tumor necrosis factor. This highlights the critical importance of early clinical recognition and the prompt initiation of immunosuppressive treatment to mitigate disease severity and reduce mortality. In contrast, delayed diagnosis and the presence of overlap syndromes were both correlated with significantly poorer outcomes and increased risk of fatal complications. ^37^

Cardiac symptoms should be managed according to the American College of Cardiology guidelines, and close monitoring in a critical care setting is often required due to the risk of rapid clinical deterioration. ^5^

Recent studies have proposed rechallenging with ICIs once symptoms revert and laboratory values normalize. However, caution is advised over this approach, and it requires careful consideration of several factors due to the risk of recurrence and severe complications. The decision should be individualized on a patient-by-patient basis, with input from a multidisciplinary team. ^5^^,^^38^^)^ The severity of myocarditis must be a key consideration, and rechallenge is typically reserved for patients with lower-grade myocarditis. The ASCO guidelines do not endorse re-initiation of therapy after grade 1 toxicity. ^5^^)^

## Prognosis and Outcomes

The prognosis of ICI-induced myocarditis is guarded, with mortality rates reported between 20-50%. ^9^^)^ Studies have suggested prognostic factors for worse outcomes, such as pre-existing autoimmune disease and higher troponin level on admission. ^16^^)^ A study conducted by Itzhaki et al. showed that severe ICI-induced myocarditis is associated with increased 1-year cardiovascular mortality. ^39^ Long-term follow-up is necessary to monitor for persistent cardiac dysfunction and to guide decisions regarding the resumption of cancer therapy.

Recent efforts to improve risk stratification in ICI-associated myocarditis have led to the development of a clinically applicable prognostic model. In a large international registry-based study, Power *et al.* proposed a composite outcome encompassing severe heart failure, life-threatening arrhythmias, respiratory failure secondary to immune-mediated myositis, and cardiomyotoxicity-related mortality. These adverse events were documented in approximately 33% of patients within 30 days of symptom onset. The multivariable analysis identified key independent predictors of poor prognosis, including the presence of active thymoma, cardiomuscular symptoms such as dyspnea or generalized weakness, reduced LVEF below 50%, low QRS voltage on baseline ECG (≤0.5 mV), and substantial elevations in troponin levels-particularly values exceeding twentyfold the upper limit of normal. ^40^

Based on these parameters, the authors developed a point-based risk score ranging from 0 to 8, which demonstrated strong discriminatory performance. This tool was externally validated in two independent cohorts from France and the United States. Notably, individuals with a score of zero experienced no major cardiomyotoxic events, suggesting that immunosuppressive therapy could be safely withheld in this subgroup. Conversely, patients with scores of four or more faced an estimated 81% risk of serious adverse events within the first month. These findings underscore the utility of this novel risk stratification instrument in guiding clinical decision-making, particularly regarding the intensity of immunosuppression and the need for close monitoring in high-risk individuals. ^40^

Cardiac irAEs may result in significant morbidity and mortality, and mortality, while interruption of oncologic therapy poses a substantial risk of disease progression and may compromise the potential for reinitiation of ICI. Accordingly, therapeutic strategies should be individualized and guided by the patient’s clinical status and a thorough assessment of cardiac function to optimize outcomes and minimize risk. ^24^ Early detection and treatment are crucial for improving outcomes; diagnostic delay and the presence of heterogeneous clinical presentation have been associated with a significantly higher risk of adverse outcomes. ^41^

## Risk Assessment

To safeguard patients from the potential adverse effects of cancer therapy, a meticulous risk assessment should stand as a cornerstone of clinical practice. Risk factors for irAEs and cardiotoxicity in general include several modifiable and non-modifiable conditions, including sex, genetic factors, past medical history, and medication history, as well as type, duration, and regimen of cancer therapies. ^42^

As established by the European Society of Cardiology guidelines, a baseline cardiac risk assessment is recommended before ICI initiation. ^35^^)^ The risk assessment for ICI-induced cardiovascular toxicity involves understanding the potential risk factors:

• Patients’ demographics: The likelihood of developing cancer therapy-related-cardiovascular toxicity (CTR-CVT) increases with age, making it a crucial factor in risk assessment. However, specifically regarding the use of ICIs, studies have shown an increased risk of severe irAE in younger populations. ^43^ Gender-based risks have also been observed, with an overall higher incidence in males, but also women on CTLA-4 inhibitors and men on PD-1/PD-L1 inhibitors have been identified as risk factors. ^(^^43^

• Medical History: Pre-existing conditions such as diabetes, hypertension, or a history of cardiovascular disease significantly elevate the risk of CTR-CVT. In addition, patients with pre-existing autoimmune diseases are at increased risk of irAEs when treated with ICIs.^44^

• Lifestyle risk factors: Smoking, poor dietary habits, and physical inactivity are well-known major contributors to overall cardiovascular risk.

• Treatment Regimen: Consider the use of mono or combination therapy to assess risk of developing irAEs. According to the European Society of Cardiology, in patients receiving ICIs, there are several baseline predictors of high or very high cardiovascular toxicity risk. Dual immune checkpoint inhibitors, combination ICI-cardiotoxic therapy, ICI-related non-cardiovascular events, or prior CTR-CVT or cardiovascular diseases are conditions that increase CVT risk. ^35^

• Baseline Tests: All patients should have baseline ECG, natriuretic peptide, troponin, and TTE, with serial laboratory and ECG monitoring in higher-risk patients. ^35^ Risk assessment should also guide referral to cardiology and cardio‐oncology specialists. These tests are critical for establishing a baseline picture of the patient’s cardiovascular health prior to initiating therapy.

## Future Directions

Further research is warranted to better understand the pathophysiologic mechanisms, risk factors, optimal monitoring strategies, and prevention of ICI-induced myocarditis. Current guidelines recommend discontinuing ICIs in patients who develop myocarditis; however, this decision can be challenging, particularly in non-severe cases where the malignancy is responding well to immunotherapy. Balancing the risks and benefits of continued ICI therapy remains a significant dilemma in clinical practice.

## Conclusions

ICI-induced myocarditis, though rare, is a life-threatening complication that requires high clinical suspicion for early diagnosis and treatment. With the increasing use of ICIs in cancer treatment, clinicians must remain vigilant for signs of myocarditis and other irAEs. Early detection through a combination of biomarkers, imaging, and clinical monitoring, coupled with prompt initiation of immunosuppressive therapy, is critical in reducing morbidity and mortality. Continued research is necessary to develop standardized protocols for managing this serious adverse event while optimizing the benefits of immunotherapy in cancer care.
